# Paddy fields located in water storage zones could take over the wetland plant community

**DOI:** 10.1038/s41598-020-71958-z

**Published:** 2020-09-09

**Authors:** Takeshi Osawa, Takaaki Nishida, Takashi Oka

**Affiliations:** 1 Graduate School of Urban Environmental Sciences, Tokyo Metropolitan University, Minami-Osawa 1-1, Hachiouji, Tokyo, 192-0397 Japan; 2 Kyoto Sangyo University, Motoyama, Kamigamo, Kita-ku, Kyoto, 603-8555 Japan; 3 Mitsubishi UFJ Research and Consulting Co. Ltd., 2-5-25, Umeda, Kita-ku, Osaka, 530-8213 Japan

**Keywords:** Biodiversity, Community ecology, Conservation biology, Wetlands ecology

## Abstract

Land use change could affect not only local species richness but also community assemblies. Essentially, the possible patterns of plant community assemblies are nonrandom species loss (nestedness) and species turnover. Plant community assemblies in human-mediated land use have a combination of both nestedness and turnover. This is because of historical effects that cause nonrandom species loss due to previous and/or original habitat quality and because of direct effects of human activities that cause species turnover. We investigated the complexity of the process of plant community assemblage in a paddy field, which is a typical agricultural land use in the monsoon season in central Japan. Using multi-temporal plant monitoring records, we tested the relationship between the ratio of species nestedness/turnover through multi-temporal and both the original habitat conditions and the extent of human modification. The findings revealed that paddy fields that originated from wetland habitat had a high nestedness ratio, whereas paddy fields that were largely consolidated had a high turnover ratio. Thus, we could divide the community assembly processes in human-mediated land use based on original habitat conditions and human activities. This concept could help land managers establish conservation and/or restoration plans that take into account community assembly.

## Introduction

Human land use impacts global biodiversity at hierarchical levels, ranging from genes to ecosystems, resulting in many ecosystems having become severely degraded^1^. Many conservation scientists are focusing on the effects of land use as factors driving habitat degradation for biodiversity and ecosystem functions and are seeking tools to counteract their degradation and loss^2–4^. Although conservation research has focused typically on either individual species or species groups in terms of their diversity and/or richness^5,6^, the impact of human activities on community assemblies goes beyond just either eradicating some species from the species pool or reducing species richness^2,7^. Habitat degradation caused by land use could cause community and metacommunity structures to collapse by inhibiting the ecological processes of assemblies^2,7^.

In essence, species loss and species turnover are the only processes required to generate all the possible patterns of community assembles^8^. Nestedness, namely, nonrandom species loss, describes the proportion of species in a species assembly that is a subset of a more species-rich assembly^2,9^. Nestedness is one of the most frequently used indices to explain patterns of community assemblages^2,10^. On the other hand, turnover can describe the proportion of species turnover in assembly processes^2,11,12^. Nestedness and turnover are antithetic (though not mutually exclusive) ecological processes that produce different patterns of community structure^8,13,14^.

Plant communities in habitat that is maintained over the long-term, for example, historical seminatural grasslands, are characterized by high species diversity and show little turnover, indicating that high multi-temporal species nestedness has occurred^15^. Thus, habitat change resulting from human activities could lead to large turnover of species; in other words, it could result in low multi-temporal species nestedness. However, although human land use change could alter the components of plant communities dramatically and inhibit their recovery^4^, plant communities can occasionally retain their components following habitat degradation, for example, through either fragmentation or reduction in area^16^. This situation is often called “extinction debt,” whereby plant species can initially survive habitat change but may subsequently become extinct without habitat modification^17^. Current plant communities in human-mediated habitats will have been established by both nested species from the original community, including extinction debt, and by species turnover from sources external to the original communities and driven by human activities. Thus, there is a complex combination of nonrandom species loss and turnover for community assemblies within human-mediated land use.

Wetlands are generally habitats with high biodiversity, and they are one of the habitats suffering the greatest decline worldwide^18,19^. Paddy fields are a typical seminatural land use for rice cropping agriculture in monsoon Asia, and they provide several ecosystem services other than rice production^20–22^. One of the important ecosystem services of paddy fields is provision of habitat for several wetland species^20,22–24^. In monsoon Asia, paddy fields originate mainly from the floodplain^24^, which is characterized by both spatial and temporal heterogeneity^25^. Floodplains provide substantial habitat variety for vegetation^26,27^. Thus, there is a variety of previous (original) habitat type/quality among current paddy fields as wetland habitat. Although paddy fields occupy the same land use category, individual paddy fields can have different types of plant communities with different assemblage processes that have been influenced both by their original habitats and by current human activities^4,24^.

We studied the complexity of plant community assemblage processes in paddy fields, which can act as alternative wetland habitats. We predicted that the importance of nonrandom species loss and turnover for plant communities in paddy fields could be assessed on the basis of both their original environmental conditions and the current human activities associated with them. In respect of original environmental conditions, we predicted that paddy fields that have originated from wetland have plant communities with a nested structure based on wetland species, because that type of paddy field is considered to be same as wetland habitat that has been maintained over the long-term. Meanwhile, paddy fields that have originated from non-wetland areas have plant communities that have undergone a relatively large species turnover from the original assemblage because that type of paddy field is considered to be habitat that has been changed from non-wetland to wetland. We used terrain condition which could indicate the surface water storage to find the original wetland potential. In respect of the current human activities, we predicted that agricultural modernization practices, land consolidation in particular, increase the species turnover because of their drastic habitat modification effects^4,28^. Previous studies have shown that land consolidation has a negative effect on biodiversity^4,28–30^. To test our hypotheses, we analyzed the relationship between multi-temporal transition of changing plant communities and location conditions namely terrain condition of paddy fields in central Japan.

## Results

In the first survey term, in 2002, there were a total of 558 species. Among these, 114 species were wetland plants, and 444 species were non-wetland plants (Table 1). In each 1-km grid, there were 89.79 ± 36.34 (mean ± S.D.) species in total, 22.09 ± 8.03 wetland species, and 67.697 ± 32.68 non-wetland species respectively (Table 1). In 2007, in the second survey term, there were 552 species in total, 109 of which were wetland plant species, 443 of which were non-wetland plant species (Table 1). There were 96.41 ± 30.59 species in total, 20.69 ± 8.17 wetland species, and 75.72 ± 26.08 non-wetland species in each 1-km grid (Table 1). In 2012, there were 469 species, 96 species were wetland plant species, and 373 species were non-wetland plant species (Table 1). In each 1-km grid, there were 72.00 ± 33.10 species in total, 18.97 ± 8.45 wetland species, and 53.03 ± 27.62 non-wetland species (Table 1).

**Table 1:** Summary of plant species numbers for each survey term.

Survey term	All species number	Wetland plants number	Non-wetland plants number
	(Mean ± S.D.)	(Mean ± S.D.)	(Mean ± S.D.)
2002	558	114	444
	89.79 ± 36.34	22.09 ± 8.03	67.697 ± 32.68
2007	552	109	443
	96.41 ± 30.59	20.69 ± 8.17	75.72 ± 26.08
2012	469	96	373
	72.00 ± 33.10	18.97 ± 8.45	53.03 ± 27.62

Generalized linear models (GLMs) analysis of the species number revealed that both field consolidation and flow accumulation values (FAVs) were negatively correlated for all species in all survey terms (Table 2). The same trend was evident for non-wetland plants (Table 2). On the other hand, wetland plants number exclude FAV values in 2012 were not significant correlations (Table 2). FAV value in 2012 for wetland plants was negatively correlated (Table 2).

**Table 2:** GLM and Wald’s test for the number of plant species in each survey term.

	Explanatory variables	2002	*p* value	2007	*p* value	2012	*p* value
		S.D		S.D		S.D	
All species	log(FAV)	−0.0138	*	−0.0154	*	−0.0206	**
		0.0064		0.0063		0.0071	
	Consolidation ratio	−0.0067	***	−0.00697	***	−0.00698	***
		0.0012		0.0011		0.0013	
	Intercept	4.7584		4.8068		4.5742	
		0.0515		0.0503		0.0567	
Wetland plants	log(FAV)	−0.0093	0.49	−0.0054	0.698	−0.0329	*
		0.0134		0.0139		0.0136	
	Consolidation ratio	−0.0026	0.26	−0.0046	0.054	−0.0011	0.66
		0.0023		0.0024		0.0025	
	Intercept	3.2282		3.1740		3.1982	
		0.1065		0.1113		0.1073	
Non-wetland plants	log(FAV)	−0.0152	*	−0.0180	*	−0.0163	*
		0.0074		0.0071		0.0083	
	Consolidation ratio	−0.0081	***	−0.0073	***	−0.0092	***
		0.0013		0.0013		0.0015	
	Intercept	4.5178		4.5907		4.2846	
		0.0589		0.0564		0.0668	

FAV indicates the sum of Flow Accumulation Values in the paddy field within the 1 km grid.

*p* value, *< 0.05; **< 0.01; ***< 0.001.

GLM analysis for species nestedness/turnover ratio revealed that both consolidation and FAV values were negatively correlated for nestedness ratios in all species for all multi-temporal combinations (Table 3). These trends were similar for non-wetland plants, excluding FAV in both 2002–2012 and 2007–2012, and the effects were not significant (Table 3). On the other hand, the nestedness ratio for wetland plants in 2002–2007 revealed a different trend; FAV was positively correlated with the nestedness ratio (Table 3; bold with italic). Other results were the same as the results for non-wetland plants (Table 3).

**Table 3:** GLM and Wald’s test for the ratio of nested plant numbers in each survey term.

	Explanatory variables	2002–2007	*p* value	2002–2012	*p* value	2007–2012	*p* value
		S.D		S.D		S.D	
All species	log(FAV)	−0.0028	***	−0.0040	***	−0.0040	***
		0.0008		0.0009		0.0009	
	Consolidation ratio	−0.0047	***	−0.00697	***	−0.0052	***
		0.0002		0.0002		0.0002	
	Intercept	4.2018		4.1112		4.1669	
		0.0063		0.0077		0.0073	
Wetland plants	log(FAV)	***0.00799***	*	−0.0019	0.60	−0.0022	0.52
		***0.0039***		0.0036		0.0034	
	Consolidation ratio	−0.0014	*	−0.00195	**	−0.0025	***
		0.0006		0.0007		0.0007	
	Intercept	2.6845		2.6185		2.7469	
		0.0302		0.0299		0.0282	
Non-wetland plants	log(FAV)	−0.0035	***	−0.0014	0.23	−0.0018	0.13
		0.0010		0.0012		0.0012	
	Consolidation ratio	−0.0055	***	−0.0083	***	−0.0062	***
		0.0002		0.0003		0.0002	
	Intercept	3.9619		3.8768		3.9163	
		0.0081		0.0102		0.0098	

FAV indicates the sum of flow accumulation values in the paddy field within the 1 km grid.

*p* value, *< 0.05; **< 0.01; ***< 0.001.

## Discussion

In this study, we tested the effects of both original environmental conditions of and current human activities in paddy fields on the processes of plant community assemblage. We found that paddy fields that originated from wetland had a high nestedness ratio of multi-temporal plant community structure during the early phase of the survey term. Also, we found that plant communities that were established in consolidated paddy fields had high turnover ratios throughout the survey terms. Thus, the findings basically supported our prediction that species community assemblages in paddy fields could be evaluated based on both the original environmental conditions of the paddy fields and the current human activities going on in them.

The study showed that plant species numbers with multi-temporal from 2002 and 2007 to 2012 were basically decreasing. From 2007 to 2012, more than 80 species, including both wetland and non-wetland plants, showed decreased numbers. This finding indicated that the basic habitat quality for plant species diversity of the paddy fields in this region had been degraded. One possible explanation of this degradation is agricultural abandonment. In seminatural ecosystems in Japan including paddy fields, abandonment has promote succession of vegetation for secondary forest which caused expanding the limited numbers of species^7,30^. Also abandonment could changes in water conditions^3,28^. Thus, agricultural abandonment could lead to a decrease of wetland and/or grassland specific plant diversity^3,7,28,30^. Over the study term, the area of agricultural abandonment in Japan increased from 3,430,000 ha (2000) to 3,960,000 ha (2010) in total^3^. The baseline of plant species diversity in paddy fields in the Tone river basin has been decreasing in recent decades.

The numbers of both all species and non-wetland plants in each survey term were negatively influenced by both FAV and the field consolidation ratio. Whereas wetland plants number was not influenced these exclude 2012 on FAV. These results indicated that areas of high FAV that can hold a large amount of water have low-quality habitat for non-wetland plants. Thus, we were able to conclude that the FAV value that we used as the index of wetland potential would be reasonable. The results regarding field consolidation were interesting because they indicated that wetland plants were not affected strongly by consolidation. Consolidation work in paddy fields had a severely degrading effect on the plant species diversity^4,28,30^. Consolidation work can not only change water conditions but also alter nutrient conditions^31^; thus, in theory, it can affect both wetland and non-wetland plants. One possible explanation for our result is that wetland plants in our study area had already recovered to a stable stage by 2002, which was the earliest term in our study. As used consolidation data for 2001, the consolidation works were conducted before 2001. Additionally, consolidation work in Japan was conducted aggressively from the 1960s to the 1990s^4^, so much of the consolidation work in the study area might have been carried out conducted more than 10 years prior to the study. Immediately following consolidation work, many plants will have been removed. However, many of plants can return and recover after consolidation work through the supply of propagules from the surrounding areas^4^. The landscapes of our study areas are dominated by paddy fields, which provide a potential source of propagules for wetland plants. Additionally seed bank of wetland plants are often prolong long time more than decades^32^. Seed bank in the paddy fields also could contribute the species recovery. Once wetland plant communities have recovered, they can be maintained in the paddy fields.

The nestedness ratio of wetland plants in the early term (2002–2007) of the study was positively influenced by FAV. This finding clearly supports our prediction that paddy fields that originate from wetland have plant communities with a nested structure of wetland species. However, on the latter term, FAV could not contribute to the nested structure of the wetland plant communities. One possible reason for this is that abandonment could have caused a decline of species in this area. Abandonment in Japan can promote succession of vegetation for secondary forest, changing the water conditions^3,7,28,30^, resulting in a decline of wetland plants in abandoned paddy fields and an increase of plants that do not prefer wetland habitats. In short, species turnover is likely to occur in abandoned paddy fields. In 2012, FAV was negatively correlated with the number of wetland plants. Also in 2012, the number of wetland plants were decreased. These suggest that succession of vegetation was in progress during this term, and thus, the wetland habitat was being degraded.

The field consolidation ratio was negatively correlated with the nestedness ratio in all cases. These findings support our prediction that consolidation could increase species turnover. Agricultural modernization, such as field consolidation, can alter the water and nitrogen conditions and the disturbance regime, and thus, is one of the main drivers of reduced biodiversity^4,7,28,30,31^. In our study, the numbers of both all species and non-wetland plant species were negatively correlated with the consolidation ratio. Therefore, consolidation cause species turnover from wetland species to limited numbers of species which adapted consolidated conditions. Uchida et al. (2018) suggested that agricultural modernization work, such as consolidation, could result in biotic homogenization; namely, decreasing beta diversity due to habitat simplification. In our study, a similar pattern may have occurred; for example, after consolidation, paddy fields as wetland habitat make similar community which could have some specific species. Although plants can recover to some extent after consolidation work^4^, consolidation is one of the main drivers of decreasing biodiversity and affects not only species diversity but also the community assembly process.

The findings of our study indicate that the plant species community assembly process in human-mediated habitats, which includes a combination of both nonrandom species loss and turnover for plant community, could estimate based on both their original environmental conditions and current human activities. Although we tested this only in paddy fields in this study, the basic concept could apply to other land areas, such as forests. For example, some parts of plantation forests might originate from primary forest and others might not. According to our theory, plantation forests that originate from long-term maintained forest could have nested community assemblies from the original community. This idea could be applied to biodiversity conservation and restoration project such as the idea of extinction debt^33^. However, estimation of the original environmental conditions presents challenges. Although a terrain component —flow accumulation value—was useful when estimating the wetland condition for paddy fields, other land uses require other methods to estimate their original habitats. If we can find a method to estimate the original habitat for not only wetland but also other habitat types we could establish conservation and restoration plans that consider not only species number but also community assembly.

## Methods

### Study site

**Figure 1:**
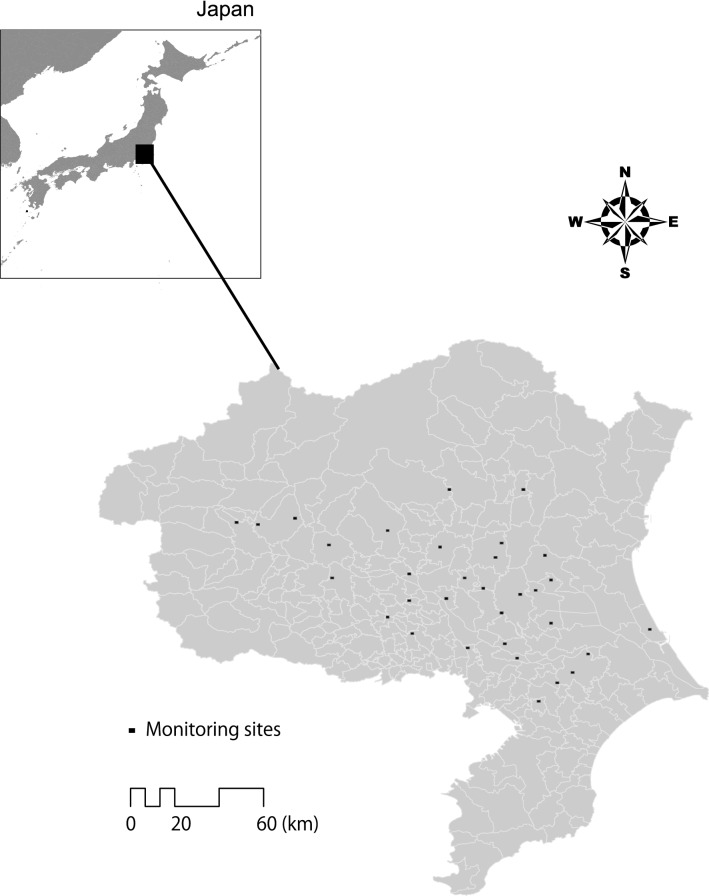
Location of the Tone river basin and monitoring sites.

The study was conducted in the Tone river basin, central Japan (Fig. 1). The Tone river is Japan’s second-longest river, running through the entire Kanto plain in central Japan. The Tone river basin is covered mainly by rice paddies and also contains arable fields other than rice, seminatural grasslands, coppice forests, farm villages, and urban areas^29^. The Tone river basin is located in the Kanto plain which is the largest plain field in Japan (approximately 170,000 km^2^), including large floodplains. Thus, this area have a variety of both terrain conditions and agricultural modernization works.

### Plants community data

The Institute for Agro-Environmental Sciences, NARO, Japan conducted the program for monitoring biodiversity, including birds^29^ and plants^34^, in each of the thirty-two 1-km^2^ grids in the Tone river basin in 2002. In this program, the Tone river basin was initially divided into one hundred 1-km square grids (hereafter, 1-km grid), and each square was classified into one of four major land use types in the region: (1) midstream paddy; (2) downstream lowland paddy; (3) plateau and valley-bottom paddy; and (4) urban fringe^29^. Then, eight grids were selected randomly as study sites from each land use type, making a total of 32 grids (Fig. 1). The grids were more than 5 km apart (Fig. 1), so they were spatially independent of each other. In this study we used the plant monitoring records from the program. In the plant monitoring program there were three terms—2002, 2007, and 2012—of vegetation survey based on the Braun–Blanquet approach in each 1 km grid ^35^. In each survey, approximately 20 quadrats measuring 1 m^2^ were placed randomly in each 1-km grid in each survey term and the coverage ratios of all plant species in four hierarchies—(1) tall tree, (2) semi-tall tree, (3) shrub, and (4) grasses—were recorded. In this study, we used only the grasses class without abundance and the presence or absence of species records in the grasses class. We pooled all the species records within each 1-km grid for analysis. All plant monitoring data are available as Open Data (CC BY 4.0) at github space own by Dr. N. Iwasaki who was the member of this monitoring program (https://github.com/wata909/RuLIS_monitoring, accessed at 25, May 2020).

### Dividing wetland plants and non-wetland plants

To test our hypothesis, we needed to divide the plants that typically grow in wetlands (hereafter, wetland plants) and those that typically grown in non-wetlands (hereafter, non-wetland plants) to evaluate the habitat quality of paddy fields as wetland. To this end, we used a published checklist of wetland plants in Japan (Shutoh et al. 2019; https://wetlands.info/tools/plantsdb/wetlandplants-checklist/, accessed at 25, May 2020). This checklist defined 8,358 Japanese vascular plants as wetland and aquatic plants according to their habitat requirements and the “wetland” definition of the Ramsar Convention (Ramsar Convention Secretariat 2016, https://www.ramsar.org/sites/default/files/documents/library/manual6-2013-e.pdf, accessed at 25, May 2020). We used this checklist to identify the wetland plant species in the monitoring records.

### Land use, terrain condition, and human activity

A digitized land use map for paddy fields in 2009 that relatively matched the plant monitoring terms (2002, 2007, and 2012) was prepared from the National Land Numerical Information (National Land Information Division, MLIT of Japan: https://nlftp.mlit.go.jp/ksj-e/index.html, accessed at 25, May 2020). These map data were developed using both topographic maps and satellite imaging data, with the land use labeled on the basis of nationwide land use classifications, including paddy fields, at approximately 100-m grid resolution (National Land Information Division, MLIT of Japan: https://nlftp.mlit.go.jp/ksj-e/index.html, accessed at 25, May 2020).

**Figure 2:**
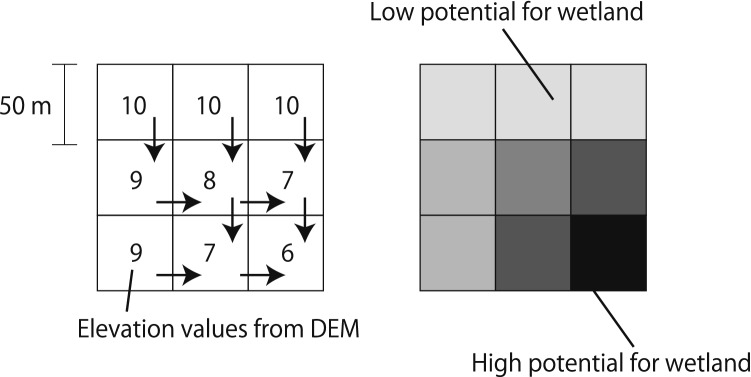
Conceptual image of the flow accumulation value to indicate the potential of wetland.

A FAV, which was ascertained by accumulating the weights of all cells that flowed into each downslope cell, was used to define the concave areas (ESRI, https://pro.arcgis.com/en/pro-app/tool-reference/spatial-analyst/how-flow-accumulation-works.htm, accessed at 25, May 2020); lower elevations and valley areas had a higher FAV because they could potentially store more water, whereas higher ridge areas had low FAVs (Fig. 2). We used FAV to define the wetland potential, as this value could reflect the water accumulation from upper areas to lower areas, which strongly relates to the natural process of wetland formation^36^. We considered that terrain variable could reflect the geographical conditions of paddy field namely potentially wetland habitat for their intact ecosystem. We considered high FAV areas to have high potential of wetland habitat for their intact ecosystem. We calculated FAV value on a whole for mainland Japan; therefore, that range could cover the entire basin which overlapped with our target areas. The FAV was calculated using ArcGIS 10.5 with Spatial analyst (ESRI, Redlands, CA, USA) using a 50-m digital elevation model from the Japanese Map Centre (https://www.jmc.or.jp/, accessed at 25, May 2020). The FAV and paddy field maps were overlaid, and the total FAVs for paddy fields in each 1-km grid were calculated to determine the potentiality of the paddy fields in the 1-km grid being wetland. If a paddy field had an extremely high FAV within the basin which included the paddy field, that paddy field could have been a wetland because that area could store a large amount of water naturally.

The proportional area of field consolidation as current human activity was calculated for each grid square using digital polygon data on the shape of farmland, as derived from aerial imagery collected in 2001 by the Ministry of Agriculture, Forestry, and Fisheries (MAFF), Japan. We obtained data on land leveling in agricultural areas from MAFF (https://www.maff.go.jp/j/tokei/porigon/, accessed at 25, May 2020) and used these data as an index of consolidated farmland because land leveling is one of the important components of agricultural consolidation in Japan^4,23^. Generally, agricultural consolidation in Japan involves land leveling, which integrates small, patchy farmland areas. Each polygon was assigned a status of “leveled” or “not leveled” according to its current status. Using ArcGIS, we calculated the ratio of consolidation for paddy fields in each 1-km grid that had survey sites.

### Statistical analysis

We performed the two types of analysis used in this study with the statistical package R version 3.5.2 (R development core Team, https://www.r-project.org/, accessed at 17, Feb. 2020). First, we tested the species number in each 1-km grid using GLM with Poisson distributions (log link) and a Wald test^37^. The response variables were total species number, number of wetland plants, and number of non-wetland plants in each 1-km grid in each survey term. Explanatory variables were the log-transformed FAV values for the paddy fields and consolidation ratio of the paddy field within the 1-km grid. The aim of this analysis was to assess the effects on species diversity of both the original environmental condition of and current human activities in the paddy fields. Prior to the GLM analysis, all explanatory variables were tested for multicollinearity by calculating the variance inflation factors (VIFs)^38^; no significant multicollinearity was found (VIF < 10 for all variables).

Second, we tested the number of both nested and turnover species from previous survey terms using GLMs with Poisson distributions (log link) and a Wald test^37^. We set three multi-temporal combinations: 2002–2007, 2002–2012, and 2007–2012. We used species number which observed both term as response variables, and used species number of latter term as offset term. Thus, we tested the ratio of species that were nested from the previous community, namely, non-turnover rates. Our analysis included all plants, both wetland plants and non-wetland plants, in each 1-km grid. Explanatory variables were the same as the species number analysis. We predicted that grids that were potentially wetland grids, namely, high FAV grids, would have a large ratio of nested wetland species. Furthermore, we predicted that heavy consolidated grids would have a large ratio of species turnover that could be negatively influenced.

## Data Availability

Data availability in this study are shown in the “Method” section.
